# A Case Report of Subacute Combined Degeneration Due to Nitrous Oxide-Induced Vitamin B12 Deficiency

**DOI:** 10.7759/cureus.34514

**Published:** 2023-02-01

**Authors:** Jorge Nadal Bosch, Javier Malcolm, Mario Moya, Michael Menowsky, Roberto A Cruz

**Affiliations:** 1 Internal Medicine, Doctors Hospital at Renaissance/University of Texas Rio Grande Valley, Edinburg, USA; 2 Medical Information, Doctors Hospital at Renaissance, Edinburg, USA; 3 Radiology, Doctors Hospital at Renaissance/University of Texas Rio Grande Valley, Edinburg, USA; 4 Emergency Medicine/Critical Care, Doctors Hospital at Renaissance/University of Texas Rio Grande Valley, Edinburg, USA; 5 Neurology, Doctors Hospital at Renaissance/University of Texas Rio Grande Valley, Edinburg, USA

**Keywords:** vitamin b12, vitamin b12 deficiency, nitrous oxide, nitrous oxide toxicity, inhaled nitrous oxide, nitrous oxide abuse, subacute combined degeneration

## Abstract

Nitrous oxide, also known as “laughing gas,” is a naturally occurring gas that is colorless, odorless, nonflammable, and nontoxic. It has been used as an inhalant anesthetic in the medical field for more than 150 years for dental and surgical procedures. Due to its wide availability and ability to cause euphoria, its recreational use is on the rise. We present a case of subacute combined degeneration (SCD) due to nitrous oxide-induced vitamin B12 deficiency. The patient presented with bilateral lower extremity paresthesia, weakness, and ataxic gait. The patient was found to have vitamin B12 deficiency. An MRI of the cervical spine revealed an abnormal T2 signal within the cervical spinal cord extending from the level of C2-C6 affecting only the posterior column. On the fifth day of hospitalization, the patient reported that he had been inhaling nitric oxide from whipped cream cans for recreational use. According to his clinical presentation and laboratory and imaging findings, we concluded that the patient had SCD. The patient slowly improved after receiving vitamin B12 supplementation therapy. Patients presenting with paresthesia, weakness, and laboratory studies indicating vitamin B12 deficiency should be questioned about nitrous oxide recreational use since the incidence is increasing.

## Introduction

Nitrous oxide, also known as “laughing gas,” is a naturally occurring gas that is colorless, odorless, nonflammable, and nontoxic. It has been used as an inhalant anesthetic in the medical field for more than 150 years for dental and surgical procedures. Due to its wide availability and ability to cause euphoria, its recreational use is on the rise [1]. Nitrous oxide interferes with the bioavailability of vitamin B12 and can cause neurotoxicity. Based on the 2016 Global Drug Survey, the lifetime prevalence of using it is 38.6% and 29.4% in the United Kingdom and the United States, respectively. In the United Kingdom, it is the eighth most commonly used substance and 29.6% of abuse occurred in the United States [2]. Here, we will present a case of subacute combined degeneration (SCD) due to nitrous oxide-induced vitamin B12 deficiency and readers will be able to recognize how important is the doctor-patient relationship and why trust is a fundamental part of the relationship. Based on the clinical presentation, neuromyelitis optica spectrum disorder (NMOSD) was high on the differential diagnosis, so the patient was started on high-dose steroids and intravenous immunoglobulin (IVIG). Even though the patient was asked about any recreational drug use on admission, he denied it until day five of admission when he admitted to using nitric oxide recreationally. This case will not only demonstrate the interesting mechanism of how nitrous oxide causes vitamin B12 deficiency but will also show how important it is to establish an excellent patient-doctor relationship to arrive at the most likely diagnosis in a timely manner. In this way, it is easier to provide high-value care providing the best care possible delivered at the right price.

## Case presentation

A 23-year-old healthy right-handed Caucasian gentleman presented to the ED complaining of one week of bilateral lower extremity symmetric paresthesia, weakness, and difficulty with ambulation. He initially experienced paresthesia in the lower extremities. Two days later, he experienced difficulty ambulating, which led to multiple falls at home. The patient stated that sensation in both legs was reduced in addition to having trouble with positioning during movement and balance difficulty. The patient also reported that decreased strength in his lower extremities bilaterally progressed within days to an inability to stand by himself and began having frequent falls, which prompted him to visit the emergency department. The patient denied trauma, fever, chills, fatigue, weight loss, nausea, vomiting, diarrhea, dizziness, and vision changes. He denied alcohol use, tobacco use, or drug use.

On neurological examination, the patient was alert and oriented to place, time, and situation, with normal five-minute recollection, normal mood, no aphasia, and cranial nerves II-XII intact. Motor strength was 4/5 in hip flexion bilaterally with eyes open, with deficit through muscular weakness. Dorsiflexion was 4/5 bilaterally with eyes open and decreased to 3/5 with eyes closed. Deep tendon reflexes were 2+ in the bicep bilaterally, 3+ in the patella with cross-adductor response bilaterally, and 3+ in Achilles, with no clonus and absent Babinski. On sensory exam, there was profound impairment in proprioception and vibration in bilateral lower extremities and decreased sensation to vibration using a 128 Hz tuning fork in bilateral upper extremities. Proprioception was preserved in the upper extremities. The patient had a normal finger-to-nose test, normal heel-to-shin test, and normal rapid alternating movements. The patient had no tremors, and he was found to have an ataxic gait.

**Figure 1:**
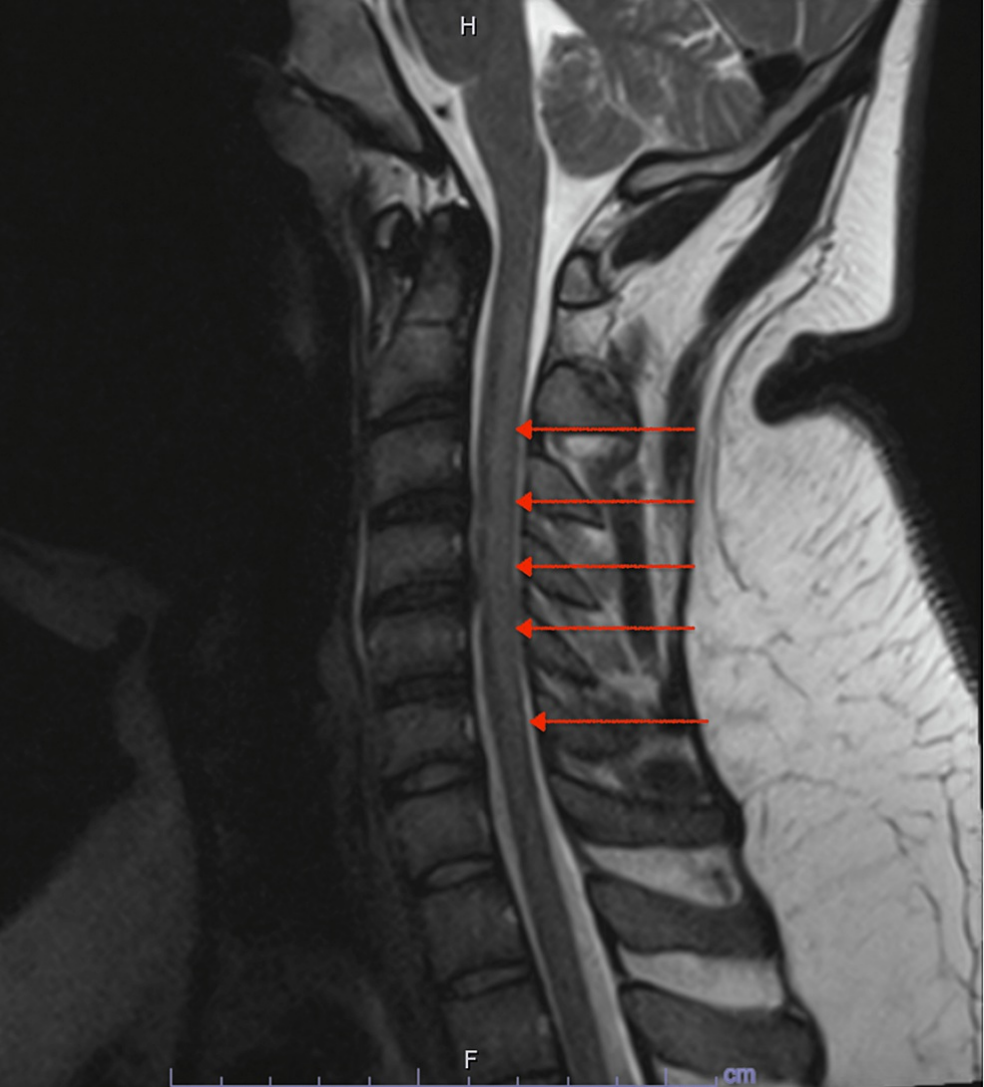
Sagittal view of abnormal T2 signal within the cervical spinal cord extending from the level of C2-C6, located in the posterior column, without associated abnormal enhancement.

Laboratory studies, including complete blood count, complete metabolic panel, thyroid-stimulating hormone, creatine kinase, lactate dehydrogenase, and folate levels, were within normal limits. Urine drug screening was only positive for tetrahydrocannabinol (THC). Serum vitamin B12 was low at 78 pg/mL and vitamin D was low at 13.9 ng/mL. MRI with and without contrast of the cervical spine showed an abnormal T2 signal within the cervical spinal cord extending from the level of C2-C6 without enhancing gadolinium (Figure 1).

The patient was started on vitamin B12 and vitamin D supplementation. Based on the clinical presentation, NMOSD was in the differential diagnosis so aquaporin 4 antibody and anti-myelin oligodendrocyte antibody were ordered. While waiting for laboratory results, the patient was started on IVIG as well as methylprednisolone IV. HIV type 1 and 2 antibodies revealed no reactivity. Immunologic workup with antinuclear antibody (ANA), double-stranded DNA (dsDNA), Sjögren's syndrome-related antigen A (SSA/Ro) antibody, Sjögren's syndrome-related antigen B (SSB/La) antibody, and gastric parietal cell antibody was negative. Lumbar puncture was performed with CSF studies showing normal opening pressure, glucose, and white blood cell count; however, CSF protein was elevated at 243 mg/dL. An infectious workup with CSF culture, CSF acid-fast bacilli, CSF venereal disease research laboratory (VDRL), and CSF West Nile virus was negative. CSF oligoclonal revealed no bands. CSF cytology was negative for malignant cells. Aquaporin 4 antibody and anti-myelin oligodendrocyte glycoprotein antibody were negative. A brain MRI to complete a demyelinating workup was also completed, which was negative for multiple sclerosis.

On day five of hospitalization, the patient completed five doses of IVIG and methylprednisolone. He also reported that he has been inhaling nitric oxide from whipped cream cans for recreational use for approximately a year. The workup revealed no evidence of infectious or immunologic underlying disease. The patient’s condition improved progressively with vitamin B12 supplementation and physical therapy, enabling him to be transferred to inpatient rehabilitation to continue recovering.

## Discussion

**Figure 2:**
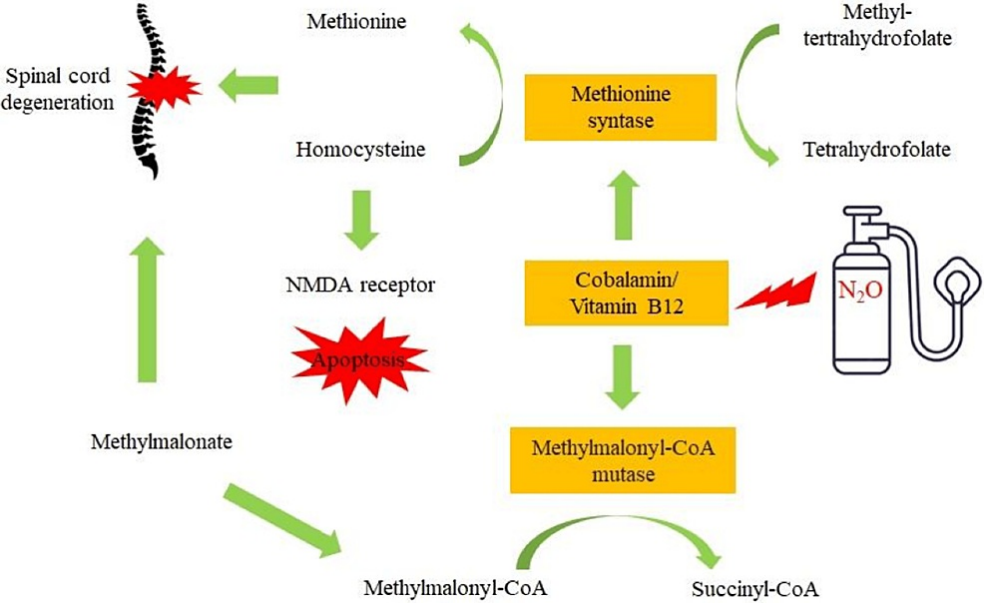
Metabolic pathway of vitamin B12 being inactivated by nitrous oxide resulting in spinal cord degeneration. NMDA: N-methyl-D-aspartate. Adapted from: Brunt TM, van den Brink W, van Amsterdam J. Mechanisms involved in the neurotoxicity and abuse liability of nitrous oxide: a narrative review. International Journal of Molecular Sciences. 2022; 23(23):14747.

Nitrous oxide causes the oxidation of cobalt ions in vitamin B12 leading to its inactivation. Inactivated vitamin B12 is unable to function as a cofactor for methionine synthase, which normally converts homocysteine to methionine. Methionine is a precursor of S-adenosyl methionine, which is used as a methyl donor required for the maintenance of the integrity of the neuron sheath (Figure 2). This will result in a damaged myelin sheath by impairing the methylation of myelin basic proteins and lipids [3].

SCD is a progressive degeneration of the spinal cord due to vitamin B12 deficiency. There are many different causes of vitamin B12 deficiency such as malnutrition, strict vegetarian diet, pernicious anemia, Crohn’s disease, celiac disease, small intestine bacterial overgrowth, alcohol abuse, autoimmune diseases, and/or medication induced. Vitamin B12 deficiency can cause a variety of neurological complications such as peripheral neuropathy, bilateral cerebral dysfunction, optic neuropathy, memory loss, personality changes, impaired recall, and SCD of the spinal cord [4]. Based on history, neurological symptoms consistent with posterior column pathology, as shown by the weakness that worsened with eyes closed, absent ankle reflexes with hyperreflexia in patellar reflexes, and profound proprioception and vibration deficit, imaging findings, and laboratory studies, the patient was diagnosed with SCD secondary to vitamin B12 deficiency induced by nitrous oxide. Patients with SCD can present with neurological manifestations such as paresthesia of the hands and feet, muscle weakness that worsens with eyes closed, sensory loss, and ataxia [5]. Treatment consists of cessation of nitrous oxide use and vitamin B12 supplementation [6]. Vitamin B12 supplementation should be started immediately upon suspected diagnosis since the response to treatment is associated with the severity and duration of symptoms. The recovery time will depend on the severity of symptoms and the degree of lesions in the spinal cord [7]. Patients should be educated on the importance of the cessation of the recreational use of nitrous oxide. Now that we were able to understand how nitrous oxide causes vitamin B12 deficiency. We should also focus on another aspect, which is the importance of establishing an excellent patient-doctor relationship and how this could impact the patient's management. If you recall when the patient was admitted, he denied any drug use but he tested positive for THC in the urine drug screening. Since NMOSD was high on the differential diagnosis, the patient was started on high-dose steroids and IVIG. The average price of one IVIG infusion is $9270. Our patient received five infusions and the total average price was $41,000. Even though we are not always going to be able to establish a great patient-doctor relationship, we should all keep in mind that we need to try our best with every patient to provide high-value care, and even if we are able to establish a perfect patient-doctor relationship with a patient, not always they will feel comfortable enough to tell personal information like drug use. For example, in this case, if the use of nitrous oxide would have been provided since the day of admission, then SCD due to nitrous oxide-induced vitamin B12 deficiency would have been first in our differential and NMOSD would have been less likely, and treatment would have consisted on vitamin B12 supplementation only without high-dose steroid or IVIG. In this manner, we would have been able to provide high-value care in a timely manner. Of note, it is very likely that the patient's condition improved due to vitamin B12 supplementation rather than the high-dose steroids and IVIG since based on clinical presentation, workup including labs and imaging pointed out to be SCD secondary to nitrous-induced vitamin B12 deficiency.

## Conclusions

Patients presenting with neurological symptoms such as paresthesia, numbness, weakness, and ataxic gait along with laboratory studies indicating vitamin B12 deficiency should be questioned about nitrous oxide recreational use. Unfortunately, there is no test to screen for nitrous oxide use, so this shows how essential it is to obtain a thorough complete history regarding recreational drug use. Recreational use of nitrous oxide is on the rise and spreading around the world. It is for this reason that all healthcare providers should be aware of this public health issue. Also, healthcare providers should always remember the importance of establishing an excellent patient-doctor relationship to provide high-value care.

## References

[1] Winstock AR, Ferris JA (2020). Nitrous oxide causes peripheral neuropathy in a dose dependent manner among recreational users. J Psychopharmacol.

[2] Kaar SJ, Ferris J, Waldron J, Devaney M, Ramsey J, Winstock AR (2016). Up: the rise of nitrous oxide abuse. An international survey of contemporary nitrous oxide use. J Psychopharmacol.

[3] Flippo TS, Holder WD Jr (1993). Neurologic degeneration associated with nitrous oxide anesthesia in patients with vitamin B12 deficiency. Arch Surg.

[4] Healton EB, Savage DG, Brust JC, Garrett TJ, Lindenbaum J (1991). Neurologic aspects of cobalamin deficiency. Medicine (Baltimore).

[5] Cheng HM, Park JH, Hernstadt D (2013). Subacute combined degeneration of the spinal cord following recreational nitrous oxide use. BMJ Case Rep.

[6] Singer MA, Lazaridis C, Nations SP, Wolfe GI (2008). Reversible nitrous oxide-induced myeloneuropathy with pernicious anemia: case report and literature review. Muscle Nerve.

[7] Jiang J, Shang X (2020). Clinical-radiological dissociation in a patient with nitrous oxide-induced subacute combined degeneration: a case report. BMC Neurol.

